# 1,8‐Cineole Protects Against Liver Toxicity Induced by Aroclor‐1254 in a Rat Model

**DOI:** 10.1002/jbt.70419

**Published:** 2025-07-28

**Authors:** Hadiqa Arshad, Muhammed Fatih Doğan, Münevver Nazlıcan Zengin, Özlem Özmen, Osman Çiftçi

**Affiliations:** ^1^ Department of Pharmacology, Faculty of Medicine Pamukkale University Kinikli Denizli Turkey; ^2^ Department of Veterinary Pathology, Faculty of Veterinary Medicine Mehmet Akif Ersoy University Burdur Turkey

**Keywords:** 1,8‐cineole, apoptosis, aroclor 1254, hepatotoxicity, inflammation, oxidative damage

## Abstract

This study aimed to demonstrate the protective effect of 1,8‐cineole against Aroclor 1254 (A1254)‐induced liver toxicity in male rats and to elucidate the underlying mechanisms. A1254 is among the persistent organic pollutants and is a toxic substance that can cause serious damage to various organs such as the liver, brain, and lungs. 1,8‐cineole, a monoterpene, possesses significant pharmacological activities such as antioxidant, anti‐inflammatory, and anticancer effects. Thirty‐two healthy young (4−6 weeks) male Wistar albino rats (200–300 g) were used. The animals were randomly divided into four equal groups: Control, Aroclor, Cineole, and Aroclor+Cineole (*n* = 8). Rats were daily administered A1254 (1 mg/kg, intraperitoneally) either alone or in combination with 1,8‐cineole (100 mg/kg orally in corn oil) for 30 days. The liver tissues and serum were collected from the rats under anesthesia. The protective effect of 1,8‐cineole against A1254‐induced liver damage was demonstrated using ELISA, RT‐PCR, histopathological analysis, and immunohistochemical evaluation methods. A1254 administration increased the total oxidant status (TOS) level and triggered oxidative stress by decreasing the total antioxidant status (TAS). However, 1,8‐cineole significantly reduced TOS levels and increased TAS levels in serum and liver tissues (*p* < 0.05). A1254 increased the pro‐apoptotic Bax gene expression in liver tissues, while 1,8‐cineole significantly decreased (*p* < 0.05). Similarly, A1254 increased the gene expressions of pro‐inflammatory cytokines tumor necrosis factor alpha (TNF‐α), interleukin‐1‐beta (IL‐1β), inducible nitric oxide synthase (iNOS), and interferon‐gamma (INF‐γ) in liver tissues, whereas 1,8‐cineole decreased the expression of these genes (*p* < 0.05). Moreover, 1,8‐cineole reduced the histopathological changes associated with A1254‐induced oxidative stress and inflammation in liver tissues. In conclusion, 1,8‐cineole may be a protective agent due to its anti‐apoptotic effect, reduction of oxidative damage, anti‐inflammatory efficacy, and amelioration of histopathological changes in liver toxicity.

## Graphical abstract

This study aimed to determine the protective role of 1,8‐cineol in Aroclor‐1254 induced liver toxicity in a rat model. 1,8‐cineol may be a protective agent due to its anti‐apoptotic effect, reduction of oxidative damage, anti‐inflammatory efficacy, and amelioration of histopathological changes in liver toxicity.

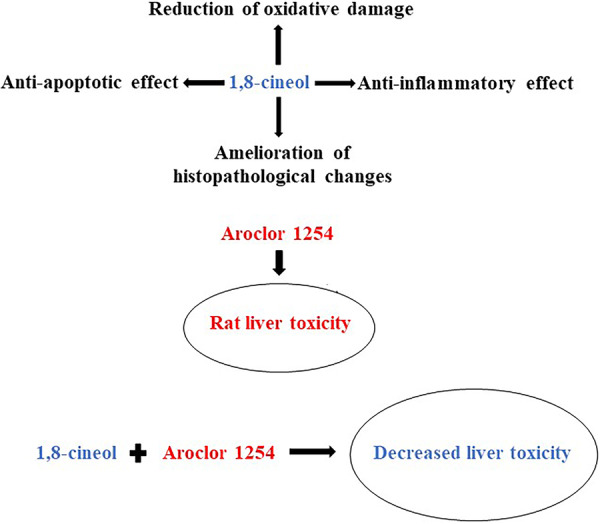

## 1 Introduction

Polychlorinated biphenyls (PCBs) are highly persistent environmental pollutants that humans are exposed to through food, water, and air, posing a potential risk to human health [1]. Much of the concern arises not only from the massive amounts of PCBs released into the environment, but also from their developmental toxicity, cancer risk, and threats to human and wildlife health. Long‐term PCB exposure causes the most harm to the human skin and liver, as well as the gastrointestinal tract, immunological system, and neurological system [2, 3].

Aroclors are commercially utilized PCB mixes with a four‐digit number, the first two of which relate to the number of carbon atoms connected to the biphenyl ring and the last two to the proportion (by weight) of chlorine [4]. This family's most often utilized compounds include Aroclor‐1254 (A1254), A1260, A1248, and A1242. Oxidative stress induced by these PCBs occurs when the equilibrium between the reactive oxygen species (ROS) and antioxidant systems is disrupted. A1254 exert its harmful effect by generating free radicals, inducing hepatic oxidative stress, thereby affecting the detoxification process [5]. A1254 induces hepatotoxicity by induction of apoptosis via upregulation of aryl hydrocarbon receptor and modulation of p53, and apoptotic protein, and upregulating the antiapoptotic protein expression patterns [6].

Terpenes, also known as isoprenoids, a diverse group responsible for plant fragrance, taste, and pigmentation, include larger classes like sterols and squalene. Terpenes are gaining commercial interest due to their potential in disease prevention, treatment, natural insecticides, antimicrobial agents, and agricultural storage as well as their potential in preventing and treating diseases like cancer [7]. As a saturated monoterpene, 1,8‐cineole is commonly used in flavored, cosmetic, or scent goods such as bath additives, mouthwashes, and insect repellants. 1,8‐cineole exhibits pharmacological activities by modulating the nuclear pathway, reducing the synthesis of ROS, and promoting anti‐inflammatory and antioxidant properties [8]. Recent studies have reported the protective effects of 1,8‐cineole on liver tissue [9, 10]. A plant such as Eucalyptol, which contains 1,8‐cineole flavonoid, has been found to have protective properties against cisplatin‐induced liver injury [11], and gentamicin‐induced kidney injury [12].

Despite the growing interest in natural compounds for hepatoprotection, the specific molecular pathways by which 1,8‐cineole exerts its therapeutic effects against Aroclor 1254‐induced liver damage, particularly regarding the interplay of oxidative stress, inflammation, and apoptosis, have not been fully elucidated. The present study aimed to investigate the therapeutic effect of 1,8‐cineole against liver toxicity induced by Aroclor 1254 in rats and the possible pathways of oxidative damage, inflammation, and apoptotic cell death, which could play a role in the mechanism of action.

## 2 Materials and Methods

### 2.1 Chemicals

Procedures were in accordance with the Guide for Care and Use of Laboratory Animals. Ethics committee approval was obtained from Pamukkale University. Aroclor‐1254 was purchased from sigma (11097‐69‐1; St. Louis, MO). 1,8‐Cineole (99%) was obtained from thermoscientific (470‐82‐6; China). Aroclor‐1254 and 1,8‐cineole were dissolved in corn oil. All the chemicals were at highest analytical grade.

### 2.2 Animals and Treatment

A total of 32 male rats, 4−6 weeks weighing 200−300 g were used in the study. Experimental animals were fed free, at room temperature, 55%−60% humidity, and 12 h of dark/light environment. The dose of 1,8‐cineole was determined based on a previous study [13]. The experimental Aroclor‐induced liver damage model was established as described previously [6].

The rats were then classified into four groups: Control group, Aroclor group, Cineole group and Aroclor + Cineol group. The control group was administered with corn oil (orally; 0.5−1 mL), cineole dissolved in corn oil was administered to the cineole group for consecutive 30 days (orally; 100 mg/kg), Aroclor, dissolved in corn oil, was administered to the aroclor group every other day (total of 10 doses given intraperitoneally; 1 mg/kg) and Aroclor + Cineole group following the former group's dose protocol. At the end of the fourth week, the rats were anesthetized, with ketamine and xylazine at respective doses of 87 and 13 mg/kg. Afterwards, intracardiac blood was drawn into serum clot activator tubes. Liver tissues were expeditiously excised and partitioned to facilitate both molecular and histological assessments. Specimens designated for biochemical and RT‐PCR analysis were promptly preserved at a temperature of −80°C in a freezer, while the remaining samples were subjected to fixation in 10% formalin for subsequent histological examination.

### 2.3 Biochemical Analysis

Blood was collected in biochemical or EDTA tubes at room temperature for 10−20 min to obtain serum, which was then centrifuged at 5000 rpm for 10 min and stored at −80° in eppendorf tubes. Tissue homogenization was performed according to Next Advance's protocol, involving weighing tissues (ideally 300 mg), cutting into smaller pieces, and homogenizing with beads in PBS at a ratio of 1:1:2 (tissue: beads: buffer) at 12 rpm for 2 min, followed by centrifugation at 15000 rpm for 5 min to obtain the supernatant. Rat TAS and TOS ELISA kits from Bioassay Technology Laboratory (Cat. No E1710Ra, Cat. No E1512Ra, respectively) were employed for serum and tissue analysis. The TAS and TOS levels in the serum and tissues were determined according to the manufacturer's instructions. Isolated after the color change in the wells, the degree of enzymatic degradation of the substrate was determined by absorbance measurement at 450 nm.

### 2.4 Histopathalogical Analysis

Liver tissues underwent a comprehensive histological preparation process, involving dehydration, xylene treatment, paraffin embedding, and sectioning. The cassettes with the formaldehyde‐fixed tissue samples were placed in the basket of the fully automatic tissue tracking device (Leica ASP300S; Leica Microsystem, Nussloch, Germany). All tissue cassettes were immersed in hot paraffin to allow it to penetrate the spaces created by removing water and fat. Following this, the tissues were removed from the device, and the blocking procedure was initiated. After routine follow‐up, liver samples were placed in a tissue embedding device (Leica Histocore Arcadia H) (Leica Microsystem, Nussloch, Germany) that had been heated the night before, and paraffin blocks were created. After the blocking process, all blocks were transferred to the tissue embedding device's cold table section. Three serial sections, 4−5 µm in thickness, were obtained using a Leica 2155 rotary microtome (Leica Microsystem, Nussloch, Almanya) and subsequently stained with hematoxylin‐eosin (HE) using Mayer's hematoxylin. These stained sections were then examined under a light microscope.

### 2.5 Immunohistochemistry

For immunohistochemical assessments, liver tissue sections on polylysine slides were oven‐dried at 45°C overnight before staining. The next day, sections underwent deparaffinization, rehydration, and were subjected to the streptavidin‐biotin complex peroxidase method using commercial kits. Bcl‐2 (Anti‐Bcl‐2 antibody [ab196495]) and Bax antibodies (Recombinant Anti‐Bax antibody [E63] [ab32503]) were applied at a 1/100 dilution from Abcam, followed by staining and washing steps. To block endogenous peroxidase activity, tissue sections were treated with 3% methanol‐hydrogen peroxide, followed by citrate buffer boiling and PBS washes. Mouse and Rabbit Specific HRP/DAB IHC Detection Kit‐Micro‐polymer (ab236466) was used as the secondary kit and 3,3´‐diaminobenzidine (DAB) as the chromogen. For negative controls, antibody diluents were replaced at the primary antibody stage. Counter staining was performed with Harris hematoxylin, and after this step, the coverslip‐sealed preparations were examined under a light microscope to evaluate the expressions. Immunohistochemical stainings were scored between 0 and 3. 0 = no staining, 1 = mild staining, 2 = moderate staining and 3 = severe staining. The obtained scores were analyzed statistically.

### 2.6 RT‐PCR Analysis

RNA extraction from hepatic tissues was conducted using Bio Basic's ONE STEP‐RNA reagent protocol and quantification using the Biotech Take three Microvolume Plates protocol were then performed, with the results obtained through raw and background subtracted absorbance data. The isolated RNA was stored at −20°C until further analysis.

cDNA synthesis from the isolated RNAs was carried out using the One Script Plus cDNA synthesis kit in accordance with the manufacturer's protocol. Synthesized cDNAs were stored at −20°C to perform Real‐Time PCR was preserved. Step One Plus RT‐PCR device, which can read a 96‐well microplate, was used for RT‐PCR analysis. Sequences of genes (Bcl‐2, Bax, TNF‐α, IFN‐γ, IL‐1β, COX‐2, and iNOS) involved in oxidative stress, inflammation, and cell death were determined on a custom basis. B‐actin was used as a reference housekeeping gene. The reverse and forward sequences of the genes were designed using BLAST (Basic Local Alignment Search Tool) software and are given in Table 1. RT‐PCR; BlasTaq 2X qPCR MasterMix kit from synthesized RNA samples was carried out following the manufacturer's protocol.

**Table 1:** Primer sequences with their length while B‐actin as reference housekeeping gene observed by RT‐PCR.

		Primer sequence	Length (Bp)
β‐actin	Forward	TGACAGGATGCAGAAGGAGA	20
	Reverse	TAGAGCCACCAATCCACACA	20
Bcl‐2	Forward	CCGGGAGATCGTGATGAAGT	20
	Reverse	ATCCCAGCCTCCGTTATCCT	20
Bax	Forward	GTGGTTGCCCTCTTCTACTTTG	22
	Reverse	CACAAAGATGGTCACTGTCTGC	22
TNF‐α	Forward	AAATGGGCTCCCTCTCATCATCAGTTC	27
	Reverse	TCTGCTTGGTGGTTTGCTACGAC	23
IFN‐γ	Forward	CACGCCGCGTCTTGGT	16
	Reverse	TCTAGGCTTTCAATGAGTGTGCC	23
IL‐1β	Forward	CACCTCTCAAGCAGAGCACAG	21
	Reverse	GGGTTCCATGGTGAAGTCAAC	21
COX‐2	Forward	AAGGGAGTCTGGAACATTGTGAAC	24
	Reverse	CAAATGTGATCTGGACGTCAACA	23
iNOS	Forward	GCATCCCAAGTACGAGTGGT	20
	Reverse	GAAGTCTCGGACTCCAATCTC	21

### 2.7 Statistical Analysis

Statistical analysis of histopathological and immunohistochemical scores in the study One‐way ANOVA test was done in the SPSS 22.00 package program. The Shapiro‐Wilk test was utilized to assess the normal distribution of the data, and the test of Levene was utilized for the homogeneity of variances. Differences between groups were determined and compared by the Duncan test. Analysis of RT‐PCR data using the 2'∆∆CT method and quantitation was done with a computer program. On the Internet‐based Gene Globe platform Volcano Plot in the “RT² Profiler PCR Array Data Analysis” program, analyses were used. One‐way ANOVA and post‐hoc Tukey in GraphPad Prism (version 7) program were used for biochemical results. Values of *p* < 0.05 were considered statistically significant.

## 3 Results

### 3.1 Biochemical Results

**Figure 1:**
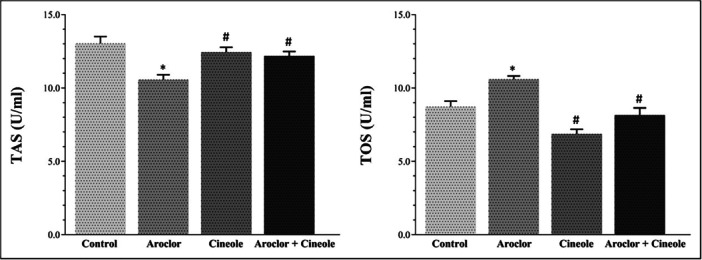
Changes in TAS and TOS levels in blood serum. *Compared to the control group; #Compared to Aroclor group. *p* < 0.05.

**Figure 2:**
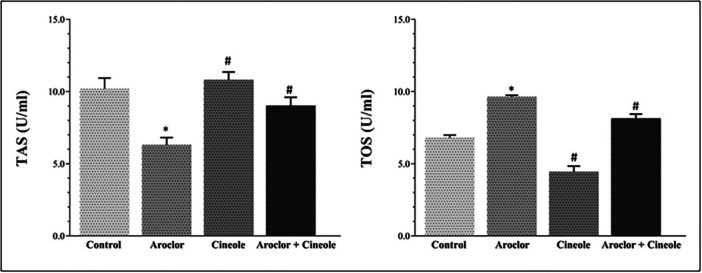
Changes in TAS and TOS levels in liver tissue. *Compared to the control group; #Compared to Aroclor group. *p* < 0.05.

Changes in TAS and TOS levels associated with oxidative damage in serum and liver tissues are given in Figures 1 and 2, respectively. Aroclor significantly increased the TOS levels and significantly decreased the TAS levels when compared to the control group in the serum and tissue (*p* < 0.05). Cineol significantly increased the TAS levels and significantly decreased the TOS levels in cineol‐administered groups when compared to the Aroclor group in the serum and tissue (*p* < 0.05).

### 3.2 PCR Results in Liver Tissue

Gene expression findings related to apoptosis, inflammation, and oxidative damage in liver tissues are given in Table 2. The fold changes of mRNA expression of Aroclor, Cineole, and A1254+ Cineol groups were compared to the control group.

**Table 2:** Gene expression results.

	Control		Aroclor		Cineole		Aroclor + cineole	
Gene	Fold change (Fc)	*p*	Fold change (Fc)	*p*	Fold Change (Fc)	*p*	Fold change (Fc)	*p*
Bax	1.00	—	3.63	0.0026*	−1.37	0.3526	1.96	0.002*
Bcl‐2	1.00	—	−1.24	0.22	−1.03	0.7843	1.08	0.5779
TNF‐α	1.00	—	7.31	0.0023*	−1.03	0.9109	3.09	0.0075*
COX‐2	1.00	—	5.81	0.017*	1.09	0.5908	3.95	0.0004*
IFN‐γ	1.00	—	2.20	0.0119*	−1.06	0.4454	1.85	0.0045*
iNOS	1.00	—	2.61	0.0088*	−1.14	0.4426	1.97	0.038*
IL‐1β	1.00	—	1.97	0.045*	−1.09	0.3786	1.55	0.0032*

Abbreviations: Fc, fold changes; Stimulation (+) and suppression (−).

* According to Control group; *p* < 0.05 was considered statistically significant.

It was determined that Bax, TNF‐α, COX‐2, IFN‐γ, and iNOS mRNA expressions were significantly increased by *3.63, 7.31, 5.81, 2.20*, and *2.61* times respectively, in the Aroclor group compared to the control group (*p* < 0.05). On the other hand, it was determined that administration of Aroclor and Cineole simultaneously increased the mRNA expressions less than the Aroclor group compared to the control. It was determined that *BCl‐2* mRNA expression decreased *−1.24*‐fold in the Aroclor group compared to the control. There was no significant change in Bax, Bcl‐2, TNF‐α, COX2, IFNγ, iNOS, and IL‐1β mRNA expressions in the Cineole group compared to the Control group (*p* > 0.05).

### 3.3 Histopathological Results

**Figure 3:**
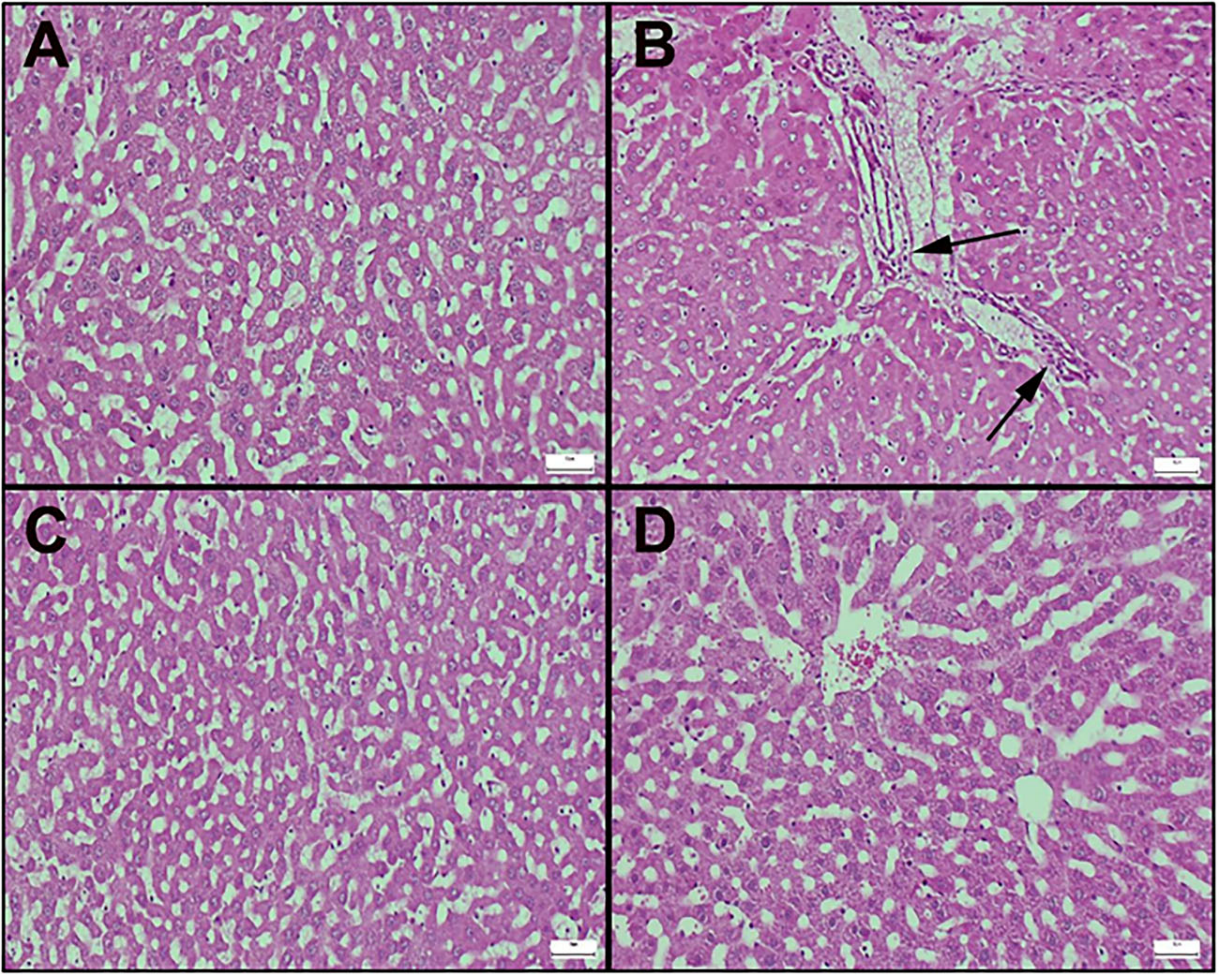
Histopathological results of liver sections; (A) control (B) aroclor (C) cineole (D) aroclor + cineole. Histopathological appearances of livers according to groups. (A) Appearance of normal liver in the control group. (B) Swelling and disruption of regular structure and inflammatory cell infiltrates in hepatocytes in the Aroclor group (arrows), (C) Significant improvement in pathological findings in the Aroclor + Cineole group. (D) Normal liver histological appearance in the Cineole group, HE, Bars = 50 µm.

Histopathological results of liver sections are shown in Figure 3. The control group shows the normal histoarchitecture of the liver (Figure 3A). The Aroclor group showed hyperemia in the vessels as well as edema, which was more prominent around the vessels. In addition, most lymphocytes and inflammatory cell infiltration, including neutrophils and leukocytes, were detected. Degenerative changes in hepatocytes were also determined (Figure 3B). Compared to the Aroclor group, the Aroclor+Cineol group exhibited reduced degenerative changes due to cineole administration (Figure 3C). The Cineole‐only group maintained a normal histological appearance (Figure 3D).

### 3.4 Immunohistochemical Results

**Figure 4:**
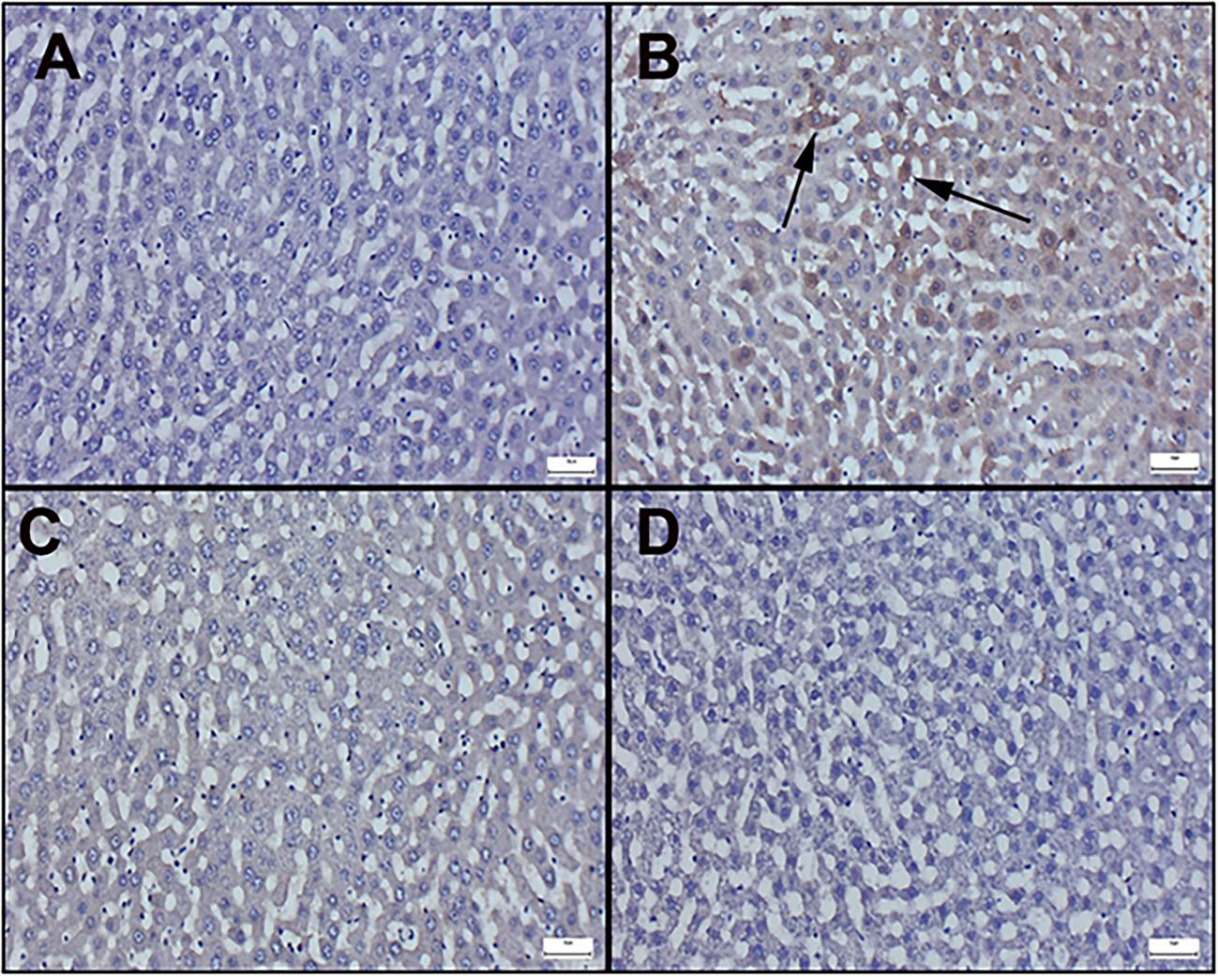
Bax immunohistochemical results of liver sections; ([A] Control [B] Aroclor [C] Cineole [D] Aroclor + Cineole). (A) Negative expression in the control group. (B) Increase in expressions in the Aroclor group (arrows), (C) Decrease in expressions in the Aroclor + Cineole group. (D) Negative expression in the Cineole group, Streptavidin biotin peroxidase method, Bars = 50 µm.

**Figure 5:**
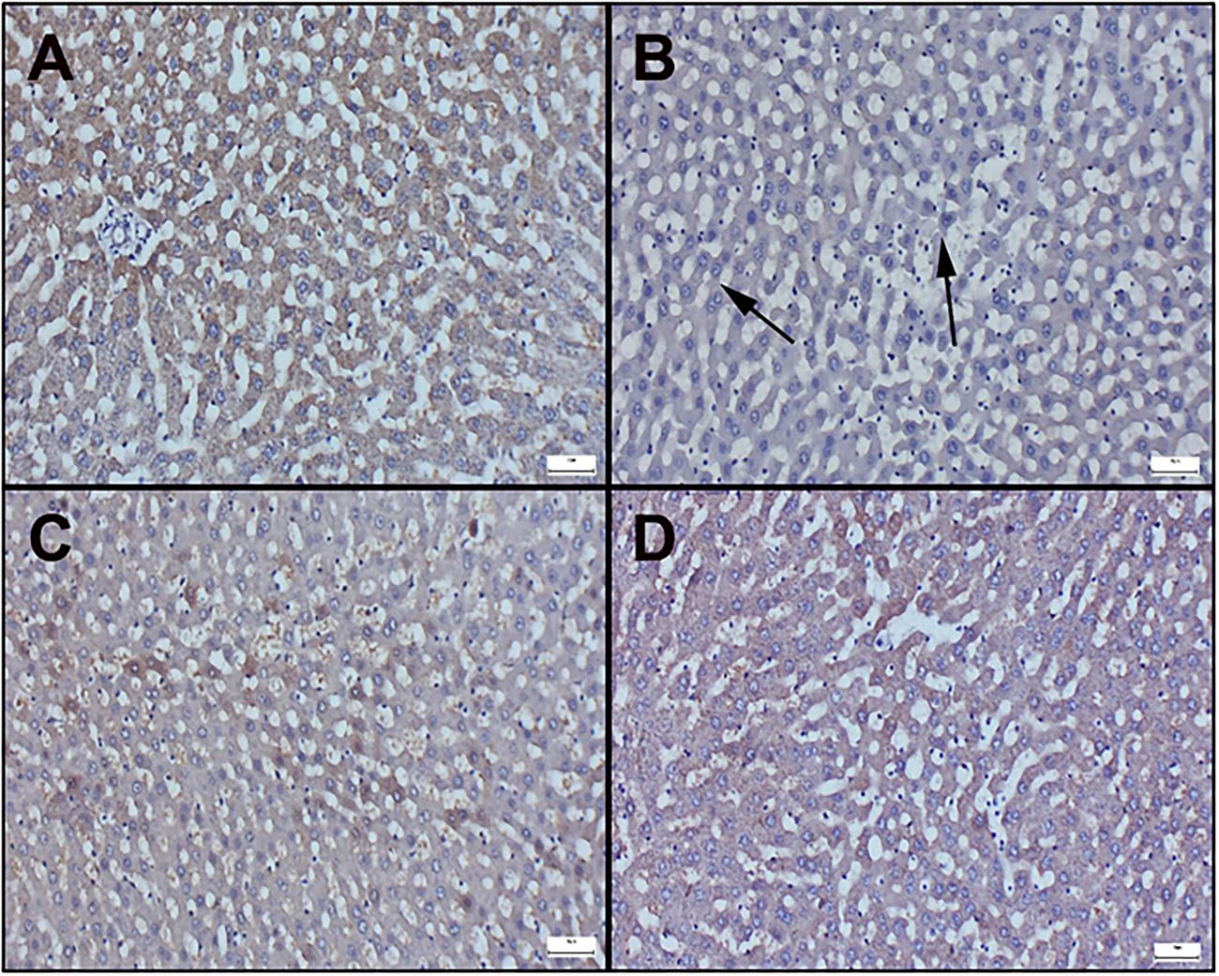
Bcl‐2 immunohistochemical results of liver sections; (A) Control (B) Aroclor (C) Cineole (D) Aroclor + Cineole. (A) Significant expression in the control group. (B) Significantly decreased expressions (arrows) in the Aroclor group, (C) Increased expressions in the Aroclor + Cineole group. (D) Significant expression in the Cineole group, Streptavidin biotin peroxidase method, Bars = 50 µm.

Immunohistochemically results of liver sections are observed in Figure 4 and 5. Additionally, immunohistochemical scores are displayed in Table 3. In the examination of Bax staining in immunohistochemically stained sections of the liver, very mild or negative expressions were observed in the control and cineole groups (Figure 4A−C). However, it was determined that the Bax expression significantly increased in the Aroclor group (Figure 4B). Cineol treatment in the Aroclor + Cineole group decreased the Bax expression, which increased with Aroclor (Figure 4D). A significant positive reaction was observed in Bcl‐2 staining in the control and cineole groups (Figure 5A−C). However, it was determined that the Bcl‐2 expression significantly decreased in the Aroclor group (Figure 5B). Cineol treatment in the Aroclor + Cineole group increased the Bcl‐2 expression, which decreased with Aroclor (Figure 5D).

**Table 3:** Statistical analysis of immunohistochemical scores.

	Bax	Bcl‐2
Control	0.14 ± 0.37^a^	2.42 ± 0.53^a^
Aroclor	2.28 ± 0.48^b^	0.71 ± 0.48^b^
Cineole	0.14 ± 0.37^a^	2.57 ± 0.53^a^
Aroclor + cineole	1.14 ± 0.69^c^	1.85 ± 0.37^c^
*p* value	< 0.001	< 0.001

*Note:* Data are given as mean ± standard deviation. Differences between groups indicated by different superscripts in the same column are statistically significant (a, b, c, d = *p* < 0.05).

## 4 Discussion

There are different possible mechanisms through which PCBs can alter normal functioning and homeostasis of the body, causing toxicity and elevated oxidative stress in different organs. Furthermore, deformation in the hepatic structural integrity is one of the signs of PCB toxicity. It was revealed that exposure to PCBs triggers an oxidative imbalance in rat liver tissues causing oxidative stress and pathological damage [14]. Aroclor 1254 is hypothesized to induce ROS formation and lipid peroxidation by stimulating cytochrome P450 enzymes [15, 16].

ROS build‐up causes oxidative stress, which is associated with the development and progression of many clinical illnesses, including inflammation, aging, and neurodegenerative diseases [17]. Aroclor may cause hepatotoxicity by initiating oxidative stress through different cellular mechanisms and inhibiting antioxidative mechanisms [18]. Previous research reported that the TAS and TOS levels in serum and liver tissues concerning the Aroclor administration significantly decreased and increased, respectively [19]. The antioxidant capabilities of 1,8‐cineole are critical to its therapeutic potential since it is excellent at neutralizing ROS and improving cellular defense systems [20]. Treatment with 1,8‐cineole increased antioxidant enzymes activities such as SOD and CAT increased total antioxidant capacity, and decreased ROS and MDA content, effectively suppressing oxidative stress [21]. We showed that 1,8‐cineole remarkably reduced hepatic and serum TOS levels while significantly increasing TAS levels in Aroclor‐induced toxicity. These findings strongly suggest that 1,8‐cineole exerts a potent antioxidant effect, possibly by restoring ROS levels to an optimal range and thereby preventing excessive oxidative stress.

It was reported that histopathological examination revealed central and peripheral necrosis, destruction of lobular architecture, and the creation of septa with sinusoidal dilatation due to Aroclor 1254 liver injury [6]. The present study showed Aroclor 1254 caused hepatocyte dysfunction, evidenced by hyperemia, vein swelling, edema, and inflammatory infiltration. However, 1,8‐cineole treatment ameliorated these effects, suggesting its antioxidant capacity may protect hepatocyte integrity against Aroclor toxicity. Immunohistochemistry indicated Aroclor 1254‐induced liver cell damage, evidenced by altered Bax and Bcl‐2 levels, while 1,8‐cineol reduced Bax levels, suggesting protection against Aroclor toxicity and implicating apoptotic cell death in the process. 1,8‐cineol's known anti‐apoptotic properties are compatible with its protective effect against Aroclor 1254‐induced toxicity.

Apoptosis has been linked to oxidative stress and ROS in numerous cell types, including hepatic cells [22]. The balance between Bcl‐2 and Bax expression regulates cytochrome c release from mitochondria, impacting susceptibility to apoptotic cell death, with increased Bcl‐2 and decreased Bax expression contributing to resistance in damaged cells [23]. 1,8‐cineole exhibits therapeutic potential by both preventing apoptosis and enhancing cell survival by inhibiting retinal apoptosis in diabetic eyes through downregulation of pro‐apoptotic markers like Bax and upregulation of anti‐apoptotic proteins such as Bcl‐2 [24]. Additionally, 1,8‐cineole mitigates isoprenaline‐induced apoptosis in H9c2 cardiomyocytes and rat heart tissue by decreasing the Bax/Bcl‐2 ratio and suppressing cleaved caspase‐3 expression [25]. Our study demonstrated that 1,8‐cineole mitigated Aroclor‐induced apoptosis in hepatocytes by reducing Bax protein levels. This suggests that 1,8‐cineole's protective mechanism involves inhibiting Bax upregulation and potentially enhancing Bcl‐2 activity. The dysregulation of pro‐inflammatory cytokines production leads to both the onset and progression of a variety of inflammatory disorders [26]. A recent study revealed that IL‐1 and TNF‐α like cytokines could facilitate dioxin's hepatotoxic effects, such as induction of hepatocyte apoptosis [27]. It has also been reported that PCB exposure in zebrafish influenced the expression of IL‐1β, IL‐8, and TNF‐α gene mRNA, which could destroy the liver, and increase the liver index [28].

1,8‐cineole effectively decreased colonic inflammation, MPO enzyme activity, and pro‐inflammatory cytokine levels, including interleukin IL‐6, IL‐1β, TNF‐α, and IL‐17A [29]. Abdollahi et al. reported that 1,8‐cineole can prevent lead acetate‐induced liver damage by mitigating oxidative stress and inflammation, likely through the inhibition of TLR4/MyD88/NF‐κB signaling [30]. Furthermore, in an acute pancreatitis mouse model, 1,8‐cineole administration resulted in lower cytokine levels of TNF‐α, IL‐1β, and IL‐6, reflecting the results found with thalidomide, a TNF‐α inhibitor [31]. The present study showed that A1254 exposure significantly increased TNF‐α and IL‐1β expression in rat hepatocytes, and 1,8‐cineole treatment decreased TNF‐α and IL‐1β expression levels. COX‐2 expression is significantly elevated during pathological processes involving toxicity and inflammation [32]. It was reported that 1,8‐cineole is a potent chemopreventive agent that inhibits UVB‐induced COX‐2 expression in HaCaT cells [33]. Our findings revealed a significant increase in COX‐2 gene expression in the Aroclor group, while 1,8‐cineole reduced COX‐2 gene expression in Aroclor toxicity.

*IFN‐γ* is a cytokine that is involved in both protective immunological responses and immunopathologic processes [34]. It has been reported that there is not a significant rise in the *INF‐γ* in the 20 μg PCB‐treated group but the rise was highly marked with the increase at 40 μg concentration of PCBs [35]. Similarly, PCBs 52 and 77 increase the amount of in the *INF*‐*γ* mouse thymocyte cultures [36]. In the present study, the significant reduction in IFN‐γ levels observed in response to 1,8‐cineole treatment against A1254 toxicity underscores its anti‐inflammatory effects. In most pathological conditions, such as liver toxicity, iNOS produces large amounts of NO, which is the main source of reactive nitrogen species [37]. It has been stated that 1,8‐cineol decreased the level of NO and down‐regulate iNOS, TNF‐α, and IL‐6 by suppressing the inflammatory responses mediated by lipopolysaccharide‐stimulated RAW 264.7 macrophages [38]. Our study showed that there is a significant increase in iNOS levels in the Aroclor group. However, the decrease in iNOS levels with 1,8‐cineole treatment indicates its potential ameliorative effect on Aroclor‐induced oxidative damage.

This study, while informative, has certain limitations. To comprehensively determine the most effective therapeutic dose of 1,8‐cineole, future studies should include a broader dose range. Furthermore, we suggest incorporating more advanced molecular methods and evaluating effects across diverse age groups and genders to fully understand 1,8‐cineole's protective and therapeutic potential.

## 5 Conclusion

In the present study, exposure to A1254 has been observed to induce liver toxicity. Simultaneously administration of 1,8‐cineole was found to reduce oxidative stress and inflammatory damage, and ameliorate histopathological injuries in liver cells. Additionally, it was determined that 1,8‐cineole exerts its protective effects through anti‐apoptotic mechanisms. Our study suggests that 1,8‐cineole would be a potential protection drug for liver toxicity exposed to Aroclor‐1254. Nevertheless, further investigations are required to elucidate the detailed molecular mechanisms of 1,8‐cineole in conferring protection against PCBs or Aroclor 1254 toxicity.

## Author Contributions

All authors contributed to the study conception and design. Material preparation, data collection and analysis were performed by H.A., M.F.D., M.N.Z., and Ö.Ö. The first draft of the manuscript was written by H.A. and M.F.D. All authors commented on previous versions of the manuscript. All authors read and approved the final manuscript.

## Ethics Statement

This study was conducted with the approval of the local ethics committee for animal experiments, Pamukkale University, Denizli, Turkey (Approval Number: PAUHDEK−2022/28).

## Consent

The authors have nothing to report.

## Conflicts of Interest

The authors declare no conflicts of interest.

## Data Availability

All data generated or analyzed during this study are included in the manuscript.
